# No evidence, no problem? A critical interpretive synthesis of the vulnerabilities to and experiences of sexual violence among young migrants in Europe

**DOI:** 10.1080/16549716.2024.2340114

**Published:** 2024-04-23

**Authors:** Tanya Andersson Nystedt, Tobias Herder, Anette Agardh, Benedict Oppong Asamoah

**Affiliations:** Social Medicine and Global Health, Department of Clinical Sciences Malmö, Faculty of Medicine, Lund University, Malmö, Sweden

**Keywords:** Migrants, youth, sexual violence, critical interpretive synthesis, systematic review, Europe

## Abstract

**Background:**

Growing evidence indicates that young migrants are particularly vulnerable to sexual violence, however most research has focused on instances of sexual violence occurring in conflict zones and during transit. Much less attention has been given to the vulnerabilities to and experiences of sexual violence among young migrants in Europe.

**Objectives:**

To understand the scientific evidence regarding the experiences of and vulnerabilities to sexual violence among young migrants (aged 11–30 years) in Europe.

**Methods:**

A search of three databases resulted in 1279 peer reviewed articles published between 2002 and 2022. Of these, 11 were included in this review. A critical interpretive synthesis methodology was applied.

**Results:**

Few studies investigate sexual violence among young migrants in Europe. The existing studies focus on very specific sub-groups of migrants, and as such, experiences of persons outside these groups are largely absent from the academic discourse. How sexual violence is understood varies across studies, often conflated with other forms of violence, hampering comparisons. However, the results of this review indicate that young migrants in Europe, both male and female, experience sexual violence and there are multiple sources of vulnerabilities at all levels of the socioecological model.

**Conclusion:**

The scarcity of research regarding sexual violence among young migrants in Europe could give rise to the perception that no evidence means no problem, resulting in a continued lack of attention to this issue. There is a critical need to address this gap to inform prevention interventions, to identify victims, and to facilitate access to care.

## Paper Context

● **Main findings:** There is a dearth of European studies investigating sexual violence young migrants’ though what little there is indicates that they are particularly vulnerable.

● **Added knowledge:** This study employed a critical interpretive synthesis compiling all available academic research between 2002–2022 identifying only 11 relevant studies.

● **Global health impact of policy and action:** More research is required to understand young migrants’ vulnerabilities to and experiences of sexual violence in order facilitate the development of appropriate prevention and response strategies that meet their needs.

## Introduction

Sexual violence is a significant public health and human rights concern leading to severe short- and long-term physical, mental, sexual, and reproductive health (SRH) consequences for its victims [1]. It also carries substantial socioeconomic implications for not only for the victims themselves but also for their peers, families, communities and society at large [2]. Prevalence of sexual violence has consistently shown to be higher among younger age groups [2] and there is increasing recognition that migrants are particularly vulnerable to sexual violence [3–5]. Young migrants face a double-burden of vulnerabilities to sexual and reproductive health and rights (SRHR) violations due to both age and migrant status. These vulnerabilities are compounded by factors such as limited or absent social and family networks, unstable living situations and difficulties in comprehending the local language and cultural context [6]. These and other factors not only make young migrants more accessible targets for potential perpetrators but also result in that they make less reliable risk assessments. There is some evidence that young migrants are particularly vulnerable to sexual violence [6,7]. However, not much attention has been given to the issue of sexual violence among young migrants in the European setting.

There is no single, internationally agreed upon definition of sexual violence. The most commonly used one is the World Health Organization (WHO) definition: ‘any sexual act, attempt to obtain a sexual act, unwanted sexual comments or advances, or acts to traffic, or otherwise directed against a person’s sexuality using coercion, by any person regardless of their relationship to the victim, in any setting, including but not limited to home and work’ [2]. Coercion is integral to the definition of sexual violence and can cover a whole spectrum of degrees of force including physical force but also psychological intimidation, blackmail or other threats – for instance, the threat of physical harm, of being kicked out from a current living situation, or sex in exchange for meeting a basic need. It may also occur when the person targeted is unable to give consent – for instance, while drunk, drugged, asleep or mentally incapable of understanding the situation [2]. As such, the definition of sexual violence covers a wide range of behaviours and different degrees of force, and the definition can vary widely between studies.

In addition to the very broad definition of sexual violence, many studies investigate it as part of a composite term covering different forms of violence. Some of the most commonly used terms are gender-based violence (GBV), sexual and gender-based violence (SGBV), violence against women (VAW) and intimate partner violence (IPV). All these terms may include sexual violence, but also physical violence, psychological violence, socio-economic violence and even harmful cultural practices [8]. GBV and SGBV are umbrella terms, while VAW specifies the victim, which in this case can only be women or girls, and IPV specifies the perpetrator, which in this case is an existing or previous romantic partner [9]. Non-partner sexual violence (NPSV) is a term that specifies the perpetrator as someone with whom the victim is not in a romantic relationship, and it refers specifically to sexual violence [10]. The use of these composite terms can obscure the experiences and effects of sexual violence or specifically neglect certain victim groups. For instance, studies on VAW exclude men and boys, while the studies on IPV overlook victims not in romantic relationships. Conversely, studies on NPSV fail to address victims in romantic relationships with the perpetrators. The experiences of men and boys in general have been largely neglected in sexual violence research, which is particularly problematic in the context of migrants, given the growing evidence of their vulnerabilities to sexual violence at all stages of migration [11].

As with sexual violence, there is no single internationally agreed upon definition of migrant. In many studies exploring sexual violence, the focus is often on specific categories of migrants, typically refugees and asylum seekers. A refugee is an individual fleeing human rights violations or persecution in their home country, while an asylum seeker is someone seeking protection on the same basis but who has not yet been granted legal recognition as a refugee and is awaiting a decision on their asylum claim [12]. Other related, and partially overlapping terms include ‘irregular migrants’, referring to migrants entering a country without a right of entry or stay and who do not possess refugee status [13] and undocumented migrants who are in a country without the right to stay. Irregular migrants can be asylum seekers or undocumented migrants, for example. In fact, undocumented migrants can originate from any category of migrant; they may have had their asylum or residency application finally denied, or they may have lost their right to residency. Some studies particularly focus on unaccompanied migrant children, who are often asylum-seeking persons under the age of 18 migrating without a parent or legal guardian [14]. However, the term ‘migrants’ encompasses various other categories, including labour migrants, spousal or relationship migrants and international students. In certain contexts, it is used even more broadly to encompass second-generation migrants, referring to the children of migrants, and sometimes even ethnic minorities [15]. In this review, the term ‘migrant’ is used to refer to individuals with migration experience, indicating they have moved from their home countries to reside in another country.

Most research on migration and sexual violence has primarily focused on instances occurring in conflict zones and during transit [5,16]. Much less attention has been given to the vulnerabilities to and experiences of sexual violence among young migrants in Europe. This represents a critical gap that needs to be addressed in order to design evidence-based sexual violence prevention and response programmes which can facilitate the participation and integration of young individuals into their new societies.

The aim of this study was to understand the vulnerabilities to and experiences of sexual violence among young migrants in Europe. The review had two objectives. The first was to understand the scientific knowledge on this topic. The second was to critically synthesise the evidence and identify emergent themes in this field.

## Methods

### Critical interpretive synthesis

A critical interpretive synthesis [17] was deemed to be the best approach to the topic of young migrants and sexual violence given the dearth of evidence on the subject and the need to include both quantitative and qualitative studies in the systematic review. Given the limited available research on experiences of sexual violence among young migrants specifically, it also allows for a more iterative process, starting with a general research question and refining it throughout the review. Although this method allows for the inclusion of grey literature, this review has focused on peer reviewed research.

The study was registered with PROSPERO, number CRD42022380737.

### Literature search and sampling

The literature search was conducted on 2 December 2022, using three databases: PubMed, Web of Science and CINAHL. Please see the Appendix for the detailed search strategy. The inclusion criteria were any peer reviewed, English language articles published after 1 January 2002, focusing on sexual violence, migrants and young persons, conducted in Europe. A 20-year period was used due to the limited research on the topic and to increase the possibility of identifying studies. No geographic focus was included in the search to ensure that relevant articles were not missed. See Table 1 for a list of the search terms used.

**Table 1.** Search terms used to identify studies for inclusion in the critical interpretative synthesis of vulnerabilities to and experiences of sexual violence among young migrants in Europe.

Sexual violence	Sexual violence; sex offences (MeSH); sexual abuse; sex abuse; sexual trauma; sexual assault; sex crimes; sexual consent; rape
Migrants	Emigrants and immigrants (MeSH); transients and migrants (MeSH); migrants; refugees; asylum seekers
Young persons	Adolescent (MeSH); young adult (MeSH); youth; teenager; young person; girls; boys; children.

**Figure 1.**
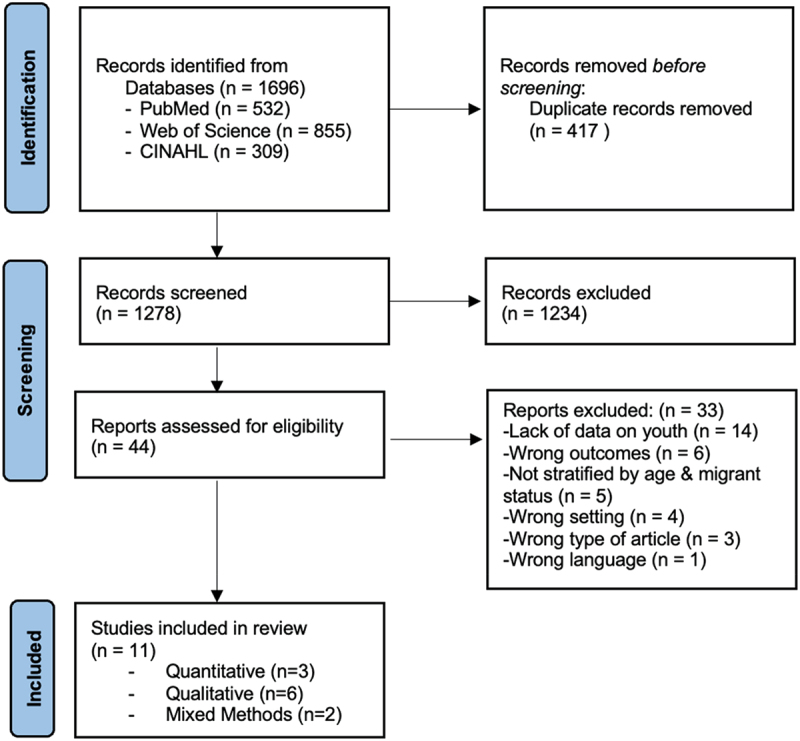
PRISMA Flow Diagram – Identification of Studies via Databases.

Search results were uploaded into Covidence for removal of duplicates, abstract and title screening and full-text review. Abstract and title screening and full-text review were carried out by two authors independently. Disagreements were discussed and consensus reached before the next step in the analysis was undertaken. Once duplicates were removed, 1279 studies were screened and 44 were selected for full-text review. Articles were considered eligible if they contained original data pertaining to sexual violence in migrants aged 11–30 years, carried out in Europe. The original age range for this review was 15–30 years, however, when undertaking the full-text review it was found not to correspond to the age ranges in the available studies. Articles that focused on trafficking were excluded as they are a very specific group of exploited people that may or may not include international migration and may or may not involve sexual violence. Studies that did not have a focus on sexual violence and migration were also excluded. Not all included studies had a focus on young persons but were included if they presented data on a relevant age group, including data from key informants. A total of 11 studies were included in the review. Backward snowballing of references from identified articles was also carried but no additional articles were included. This process was documented in accordance with the international PRISMA standard (Figure 1).

### Data extraction and synthesis

The following data were extracted from all included studies: 1) names of all the authors; 2) publication year; 3) location of the study; 4) aim of the study; 5) study design; 6) description of study population; 7) recruitment method; 8) sample demographics (age range; sex); 9) how sexual violence is defined and/or operationalised; 10) all findings relevant to young migrants (aged 11–30 years).

Each study was first understood in relation to itself. In this process, concepts, themes and relevant findings were identified. In the next step, these were then translated into the other studies and synthesised into an integrated account of the evidence through an iterative process.

### Quality assessment using the mixed methods appraisal tool (MMAT)

Quality assessment of the included studies was carried out using the MMAT [18], a tool developed to enable the methodological appraisal of quantitative, qualitative and mixed methods studies for systematic reviews. For each included study an appropriate study category was selected, which for this review included qualitative studies, quantitative descriptive studies and mixed methods studies. Each category contained five methodological quality criteria questions with possible responses ‘yes’, ‘no’ or ‘can’t tell’. Scoring is discouraged using this tool; instead, the appraisal should be used to inform the quality of the included studies [18].

## Results

### Overview of studies

Eleven articles were included in the review. Three were quantitative studies, six were qualitative studies and two were mixed methods studies (see Table 2 for a summary of the included studies).

**Table 2.** Summary table of studies included in the critical interpretative synthesis of vulnerabilities to and experiences of sexual violence among young migrants in Europe.

Included studies	Country	Type of study	Age group*	Migrant group	Location of violence
Baraoudi et al. 2021	Sweden	Quantitative	16–29 years	Born outside Sweden	Not specified
Belanteri et al. 2020	Greece	Quantitative	11–30 years (and older)	Migrants and asylum seekers	All stages of migration
Chynoweth et al. 2020	Italy (and others)	Qualitative	15–24 years (and older)	Asylum seekers and refugees	Europe
Digidiki & Bhabha 2018	Greece	Qualitative	<18 years	Unaccompanied migrant children	Europe
Jiminez-Lasserrotte et al. 2020	Spain	Qualitative	18–30 years (and older)	Irregular Migrants	Pre-migration and in transit
Keygnaert et al. 2015	Belgium, Greece, Hungary, Ireland, Malta, the Netherlands, Portugal	Mixed methods	12–29 years (and older)	Asylum seekers and refugees	Europe
Keygnaert et al. 2012	Belgium & the Netherlands	Qualitative	<18–29 years (and older)	Asylum seekers and refugees	Europe
Lay & Papadopoulos 2009	UK	Mixed methods	<18 years	Underaged asylum seeking minors	Europe
Longobardi et al. 2017	Italy	Quantitative	16–17 years	Unaccompanied migrant minors	Pre-migration and in transit
Lopez-Domene et al. 2019	Spain	Qualitative	18–30 years (and older)	Irregular Migrants	Pre-migration and in transit.
Thomas et al. 2004	UK	Qualitative	12–18 years	Underaged asylum seeking minors	Pre-migration and in transit.

*The different age limits reflect the age categories of the included studies.

#### Quality of the studies

The quality of the included studies was deemed to be generally high and appropriate to the research questions under investigation. Reflecting the difficulty in conducting research on vulnerable young migrants in Europe and the lack of evidence regarding exposures to sexual violence in this group, the majority of included studies in this review were qualitative or mixed methods and all the quantitative studies included were descriptive.

One of the key issues with the quantitative studies included in this review was the sample sizes which varied widely between 1773 [19] and 19 [20] (see Table 3 for details). Small sample sizes may be a direct consequence of investigating very specific populations in a particular context but can limit the generalisability of the data collected. Conversely many of the qualitative studies had comparatively large sample sizes ranging from 23 participants [21] to 100 participants [22].

**Table 3.** Sample sizes and the percentage of male/female/other respondents in the studies included in the critical interpretative synthesis of the vulnerabilities to and experiences of sexual violence among young migrants in Europe.

Study	N=	11–30 years	KIIs*	Male	Female	Other
Baroudi et al. 2021	1773	1773		**65%**	35%	2%^a^
Belanteri et al. 2020	215	130		30%***	70%***	
Chynoweth et al. 2020	10**	7**	148	**100%**	0%	
Digidiki & Bhabha 2018	24	0	24	n/a	n/a	
Jimenez-Lasserrotte et al. 2020	26	17		0%	**100%**	
Keygnaert et al. 2015	557	254		60%***	40%***	
Keygnaert et al. 2012	223	117		43%***	57%***	1%^b^
Lay & Papadopoulos 2009	53	53		4%	**96%**	
Longobardi et al. 2017	19	19		**95%**	5%	
Lopez-Domene et al. 2019	23	8		0%	**100%**	
Thomas et al. 2004	100	100		59%	41%	

*KIIs – Key Informant Interviews – not data collection from the target group.

**Focus Group Discussions involving an average of 6 participants each.

***Gender differences reflect the proportions in the study as a whole and not of the relevant age group as this data is lacking.

^a^Study included data on non-binary gender identities.

^b^Study included data on transsexual gender identities.

One of the studies was based entirely on data from key informant interviews with service providers and not with the target group themselves [23], which can impact the validity of the findings as they represent the participants’ perceptions of young migrants’ lived realities.

Lastly, there is a marked gender imbalance in the included studies with some studies focusing exclusively or predominantly on females while others focusing exclusively or predominantly on males. In interpreting these studies, it is important to note that the results may not be applicable to young migrants of the under-represented or absent sex.

#### Data collection

Two studies gathered data from service providers or other key informants who were not part of the target group. The study by Digidiki & Bhabha [23] was based on interviews with key informants working with unaccompanied migrant children. Chynoweth et al. [24] also included key informant interviews in addition to the 7 focus group discussions with the target group. The rest of the studies focused on very specific sub-groups of migrants (see Table 2). For example, Belanteri et al. [25] investigated migrants and asylum seekers accessing healthcare services for sexual violence at specific clinics in Greece. Another example can be found in the study by Longobardi et al. [20], which focused exclusively on young migrants living in small residential care homes in Northern Italy. This sample was largely dominated by male respondents in their late teens. This also reflects that most unaccompanied migrant children are young males.

### The state of research

There were few studies that investigated the vulnerabilities to and experiences of sexual violence among young migrants in Europe. The studies that exist focused either on very specific groups of migrants, such as refugees and asylum seekers, or on a particular gender, or both, resulting in experiences of persons outside these groups being largely neglected. Very few studies focused on young people as a group, tending to focus either on children (under 18 years) or adults (over 18 years), and in the case of adults, most studies did not disaggregate by age in all analyses.

#### Types of migrants

The included studies explored the vulnerabilities to and experiences of sexual violence among young migrants in Europe. The vast majority, 10 out of 11, focused on asylum seekers, refugees or irregular migrants and 4 of these 10 studies focused specifically on unaccompanied migrant children. This means that the experiences of any young migrants not falling into one of these four categories are largely missing from academic discourse. This is supported by the single study that looked at migrants more broadly [19], which included all migrants regardless of their reason for migrating or their legal status. This study found that vulnerabilities to sexual violence varied by sexual orientation and gender identity, legal status and region of origin [19].

#### Age

Research looking specifically at experiences of young migrants is scarce. Only one study looked specifically at young people focusing on migrants aged 16 to 29 years [19]. Four studies looked only at the experiences of children under the age of 18 years and six studies stratified by age and included older age groups (over 30 years) with varying lower age limits. This review includes data from primary studies with age-disaggregated analyses presenting information on migrants aged 11 to 30 years. However, not all the analyses in the included studies were disaggregated by age and these findings are therefore excluded from this review. The results were not applicable specifically to young migrants.

#### Sexual violence

Few of the included studies focused exclusively on sexual violence. One study focused on sexual rights where sexual violence falls under the right to bodily integrity [19]. Five studies included sexual violence among other forms of violence [5,13,20,22,26], and two studies focused on access to services for survivors of sexual violence [21,24]. All included studies used different definitions or investigated different forms of sexual violence and had different ways of operationalising it, as indicated in Table 4. These different understandings of sexual violence complicate comparisons across studies, especially when they encompass a wide range of behaviours from unwelcome comments to rape and involve varying degrees of associated violence. Broader definitions of sexual violence are more likely to identify a greater number of cases as they include more types of behaviours.

**Table 4.** Different definitions and means of measuring sexual violence in the studies included in the critical interpretative synthesis of the vulnerabilities to and experiences of sexual violence among young migrants in Europe.

Study	Understanding, forms and/or operationalisation of sexual violence
Baroudi et al. 2021	Sexual rights, the right to bodily integrity, free from coercion and violence. Measured by experiences of specific behaviours that are components of sexual violence such as unwanted sexual remarks, exposure of body parts, touching of sexual organs, harassment through the internet, vaginal intercourse, etc.
Belanteri et al. 2020	Rape, forced prostitution, sexual touching and associated violence
Chynoweth et al. 2020	Sexual violence - undefined - focused on access to services for sexual violence.
Digidiki & Bhabha 2018	Child sexual abuse: “actual or threatened physical intrusion of a sexual nature, including inappropriate touching by force or under unequal or coercive conditions”. Child sexual exploitation: “any abuse of a position of vulnerability, differential power or trust for sexual purposes; this includes profiting monetarily, socially or politically from the sexual exploitation of another.
Jimenez-Lasserrotte et al. 2020	Sexual and reproductive control including forced pregnancy and forced abortions, rape, forced prostitution and human trafficking.
Keygnaert et al. 2015	Sexual and gender-based violence (SGBV) including emotional, physical, sexual and socio-economic violence
Keygnaert et al. 2012	Sexual and gender-based violence (SGBV) including emotional, physical, sexual, and socio-economic violence and harmful cultural practices.
Lay & Papadopoulos 2009	Sexual maltreatment consisting of: 1) sexual assault (sexual maltreatment involving physical violence); 2) sexual abuse (sexual maltreatment not involving physical violence; and 3) sexual harassment (verbal or other taunts of a sexual nature)
Longobardi et al. 2017	Sexual abuse: (many times, sometimes or never) a) touched your body in a sexual way that made you uncomfortable (on genitals or breasts); b) showed you pictures, magazines or movies of people or children doing sexual things; c) made you take your clothes off for a non-medical reason; d) made you have sex with them; e) made you touch their private parts when you didn’t want to; f) gave you money or gifts to do sexual things; g) involved you in making sexual pictures or videos; h) kissed you when you didn’t want to be kissed.
Lopez-Domene et al. 2019	Sexual exploitation; rape, trafficking
Thomas et al. 2004	Rape

### Young migrants’ experiences of and vulnerabilities to sexual violence

The results of this review indicate that young migrants, both male and female, are vulnerable to and experience sexual violence. This section presents a synthesis of the findings from the included studies.

#### Experiences of sexual violence

The included studies indicate that young migrants are at risk of experiencing sexual violence at all stages of migration. Five studies focused on sexual violence that took place in Europe, four focused on pre-migration and transit, one on all stages of migration and one study did not specify where the sexual violence took place (see Table 2).

Few of the included studies reported prevalence data on sexual violence among young migrants. The different studies focused on different groups of young migrants and comparisons should be interpreted with caution. The study that was more inclusive of different migrant groups was the Baroudi et al. [19] study which reported a 25.2% prevalence of any type of sexual violence among young migrants aged 16–29 years with a slightly higher prevalence among young men (26.2%) than young women (20.7%). In fact, the included studies confirm that young male migrants are vulnerable to sexual violence.

There is some indication that being young increases the risk of being exposed to sexual violence. The Keygnaert et al. [26] study found that refugees and asylum seekers under the age of 30 years had higher odds (though not statistically significant) of having been exposed to sexual violence (OR = 2.13; 95% CI 0.17–26.03) as compared to those over 30 years. They also had higher odds (also statistically insignificant) of knowing someone exposed to sexual violence (OR = 1.88; 95% CI 0.39–8.99). The Belanteri et al. [25] study focused exclusively on refugee and asylum-seeking survivors of sexual violence who sought healthcare services. Among them a majority, 61%, were under 30 years of age, with 20% aged between 11 and 20 years and 41% aged between 21 and 30 years.

The included studies also indicated that some groups of young migrants may be more vulnerable than others. In the Baroudi et al. [19] study, the groups of young migrants reporting highest exposure to sexual violence were non-binary persons (44.8%), sexual minorities (36.8%), those awaiting decisions on their residency permits (39.9%) and those born in South Asia (30.8%).

The prevalence of penetrative sexual assault (rape) varied widely between studies (see Table 5). The Baroudi et al. [19] study reported prevalence rates of 6.5% for forced vaginal sex. The three other studies that reported prevalence rates for rape (ranging between 8% and 58.5%) focused on unaccompanied migrant children, a group that is potentially especially vulnerable. The study by Thomas et al. [22] found a prevalence rate of rape of 30% (58.5% among girls and 15% among boys). The study by Longobardi et al. [20] reported a prevalence rate of rape (defined as forced sex) of 26.3% in a sample of mostly male unaccompanied migrant children residing in care homes, while Lay and Papadopoulos [27] reported an 8% prevalence of rape among their primarily female respondents.

**Table 5.** Prevalences of rape reported in the studies included in the critical interpretative synthesis of the vulnerabilities to and experiences of sexual violence among young migrants in Europe.

Study	Study Population	Prevalence of rape
Baroudi et al. 2021	Migrants aged 16–29 years	6.5%
Thomas et al. 2004	Unaccompanied migrant children	30% (58.5% females; 15% males)
Longobardi et all. 2017	Unaccompanied migrant children	26.3% (1 female; 18 males)*
Lay & Papadopoulos 2009	Unaccompanied migrant children	8% (50 females; 2 males)*

*These studies have very skewed sex distributions and do not disaggregate prevalence of rape by sex.

Other forms of sexual violence described in the studies include sexual harassment; being shown sexual images, films or multimedia; being touched sexually against one’s will; being made to undress; being made to touch someone else’s ‘private parts’; and being kissed against one’s will. Other more brutal forms of sexual violence include an attempted rape [27], being forced to perform sexual acts for money [20] or rewards [5,13] and being forced to sell sex [5,13]. Multiple perpetrator (gang) rape was reported by both males and females [5,22] as was being raped in front of one’s family members [22].

In addition to the incredibly high exposure to sexual violence there is evidence from multiple studies of repeated exposures, where between 14% [22] to 73% [27] of respondents reported being exposed at least twice over a period of weeks, months or even years.

#### Disclosures of sexual violence

Disclosures of sexual violence were low with non-disclosure rates ranging from 33.3% [27] to 63.6% [19]. In these studies, non-disclosure referred to participants who had experienced sexual violence who had reported not having disclosed it to anyone previously. Some reasons discussed by Lay & Papadopoulos [27] were fear of perpetrator retaliation, fear of being blamed or not believed, having no one to tell and lacking trust in that professionals would respond. Lack of professional response was also touched upon by Digidiki & Bhabha [23], who suggested that young migrants felt that there was no point in disclosing. However, Belanteri et al. [25] proposed that the provision of mental health services can increase disclosure rates as sexual violence would not need to be disclosed upon entry into services and trust can be built up with the service provider.

In relation to young men and boys in particular, Chynoweth et al. [24] reported cases of self-blame for the experience of sexual violence, that something they did attracted the perpetrator, as well as a fear of being ‘turned gay’ or of no longer being a man. They also reported that sexual violence among young men and boys is also less likely to be disclosed as the stigma may affect the entire family, resulting in their ostracism from the community [24].

#### Vulnerability factors

**Figure 2.**
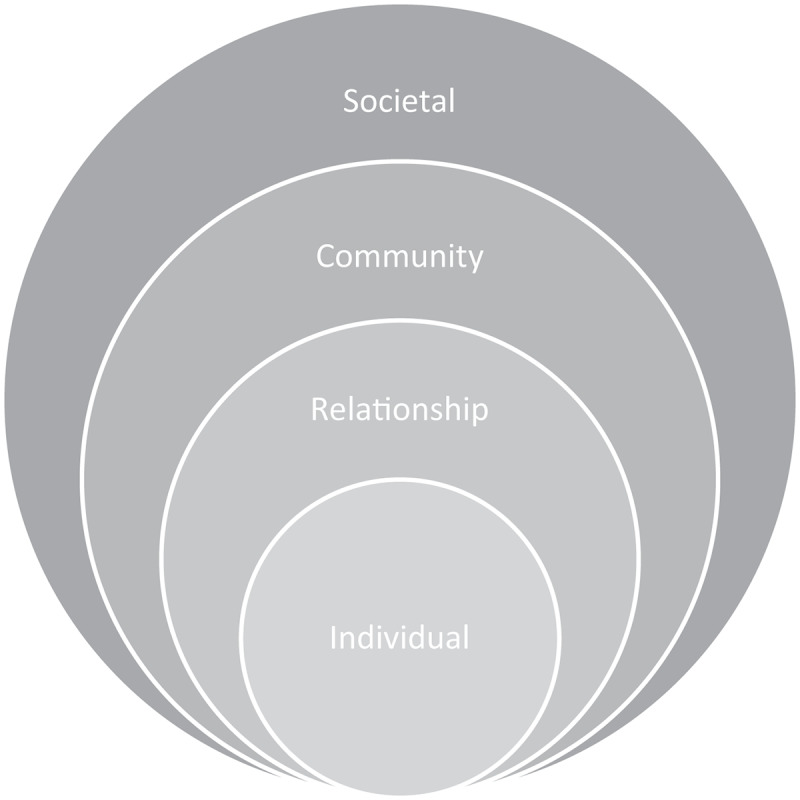
The socio-ecological model adapted from Heise [28].

Most of the vulnerability factors discussed in the included studies pertain particularly to asylum seekers and refugees [25], unaccompanied migrant children [23,27] and female irregular migrants [13]. Vulnerability factors are presented in relation to the different levels of the socioecological model adapted from Heise [28] (see Figure 2). Factors at different levels of the socioecological model may be interrelated and either influence or are influenced by factors at other levels. It should be noted that the classification of factors under the different levels of the socioecological model is based primarily on the scope and influence of such factors, as indicated by the findings of various studies and our synthesis of them. This classification does not necessarily refer to whose responsibility it is to bring about change, which could potentially fall at different levels for any given factor.

At the societal level, different migration policies hindering the access to legal and social rights for persons lacking residence permits impacts young migrants’ vulnerabilities to sexual violence. Lengthy, bureaucratic processes for gaining residency increases the amount of time they spend in these vulnerable situations [5,19]. For example, legislation around the EU relocation and family reunification scheme, the only legal pathway for onward migration for unaccompanied migrant children, has undergone substantial delays, sometimes leaving young migrants stranded in transit countries indefinitely [23]. This again increases their vulnerability to trusting others who appear to offer them a solution, such as smugglers [23].

At the community level, one of the key issues mentioned as increasing the risk of sexual violence was a lack of appropriate and secure accommodation for young migrants, particularly those travelling alone [23,27] as well as a lack of systematic security in migrant camps and facilities [23].

Economic hardship was also raised as a vulnerability factor for sexual violence [5,13,21]. Young women who migrate from their homes, often in sub-Saharan Africa, are many times expected to send money back to their families either due to their own poverty or because they helped to pay for part of the journey [13]. This also increases their vulnerabilities to trafficking and sexual exploitation.

At the relationship level, a lack of trustworthy adults [27] was raised as a risk factor for sexual violence, as was the subsequent reliance on the ‘wrong’ people who appear to want to help [5]. This can be compounded by the cultural stigma surrounding sexual violence which can contribute to the lack of awareness and therefore vulnerability to it. In addition, gender norms of being passive, shy and trusting for girls were discussed as a factor which can increase vulnerability to sexual violence [27].

At the individual level, lack of awareness of sexual violence was identified as a particular risk factor [5,24,27] as well as a lack of knowledge about their rights [27]. Loneliness and isolation were also been raised as risk factors that could make young people more likely to trust people that they should not, rendering them more vulnerable to grooming [27]. The Digidiki and Bhabha [23] study focused on factors relevant to unaccompanied migrant children living in migrant camps, but which could be applicable to asylum seeking children generally. One factor they discussed was forced passivity, a result of information not being shared with the young person and decisions being made over their heads. These young persons can find themselves in a holding pattern, sometimes for years, waiting for resolutions to their asylum processes, family reunification or onward migration, during which their own goals and personal developments are stymied. This can lead to a lack of trust in the official process, which can be compounded by having been let down by authorities in the past. This forced passivity and lack of trust can cause young migrants to attempt other means of fulfilling their goals, including engaging with smugglers, traffickers and the drug trade, all of which increase their risk of sexual violence [23].

#### Consequences of sexual violence

A few studies discussed possible outcomes of sexual violence with varying levels of severity. Keygnaert et al.. (2012) reported several including emotional-psychological distress which may result in victims isolating themselves and an inability to trust others [5]. This isolation can lead to socio-economic consequences including loss of employment or being unable to continue with their education [5]. Other reported consequences included being separated from partners and children or being rejected by their communities [5]. In extreme cases victims of sexual violence can experience fatal outcomes, either through suicide or as a direct result of the sexual violence. One reported case was of a young male migrant dying shortly after participating in the study as a result of HIV contracted from the experienced sexual violence [5]. SRHR consequences reported were unwanted pregnancies and abortions [5,13] as well as unsafe abortions and resulting infertility [5].

#### Services for sexual violence

The above mentioned consequences highlight the need for services for young migrants exposed to sexual violence. This includes SRHR and emergency services especially for newly arrived irregular migrants [21], while Belanteri et al. [25] called more generally for physical and mental healthcare services, many of which are often not available or accessible for migrants in their host countries, depending on their legal status [6]. Digidika and Bhabha [23] raise the need for properly trained trauma personnel in migrant camps. Even where they are available, the conditions are not conducive for trauma care, which takes time and requires a sense of trust, security and predictability, none of which are present [23]. In addition, when sexual violence services are available, they are usually provided in a context of what is traditionally considered women’s healthcare services, creating an additional barrier to access for men and boys [24].

#### Perpetrators of sexual violence

The included studies had a very limited discussion of perpetrators of sexual violence against young migrants. Of particular note were the high rates of multiple perpetrator sexual violence (gang rape) [5,22,27]. Otherwise, the reported perpetrators seemed to depend very much on who the victims were. In one study on mostly female unaccompanied asylum-seeking children living in reception centres, most of perpetrators were male, most often from an ethnic minority, and some were thought to be asylum seekers or refugees, sharing accommodation with their victims [27]. In other cases, the perpetrators included individuals who were in positions of authority, including those assigned to their protection [5] or carers of the young migrant, in the case of female perpetrators [27]. However, there were also a large number of perpetrators who were unknown to their victims [5].

#### Prevention of sexual violence

Lay and Papadopoulos [27] highlighted the importance of informing young people about sexual violence to increase the likelihood of both disclosure and seeking services. This was supported by Keygnaert and colleagues [5] who also underlined the critical importance of trustworthy relationships for the wellbeing of young migrants and the need to enhance social networks for prevention of sexual violence. At the organisational level, facilitating access to services for young migrants that are both safe and trustworthy was also identified as a critical intervention [5] although there are limited care options if a long time has elapsed since the victimisation [25]. At the social level, Belanteri et al. [25] called for a more preventive legislative framework increasing the rights that young migrants have and are able to access.

Digidiki & Bhabha [23] discussed gaps in the prevention of sexual violence as reported by key informants working with unaccompanied migrant children. A core issue is the lack of reliable evidence on sexual violence and the perception that this lack implies the absence of a problem, creating a vicious cycle where the identification of victims is not prioritised. In cases where victims are identified, there is a lack of coordination between different actors resulting in slow referral pathways that leave children in vulnerable situations while the abuse is being verified.

## Discussion

To the best of our knowledge, this is the first systematic review exploring the vulnerabilities to and experiences of sexual violence among young migrants in Europe. As expected, there was very little research focusing specifically on young migrants (aged 11–30 years) in Europe. Even after including studies that did not focus particularly on young migrants but included data on them, we only identified 11 peer reviewed studies over a period of 20 years. Despite this lack of research, what evidence there is indicates that young migrants, both male and female, are vulnerable to sexual violence.

The dearth of data on sexual and gender-based violence in migrant populations has been described in previous research on children [7] and adults [3,16] and is confirmed in relation to young migrants in Europe by this review. The predominance of qualitative methods found in this review can be explained partially by the sensitive nature of the topic, as such methods can be used to build trust and develop a rapport, allowing participants to put their experiences into a context and share their stories [3]. It may also be due to the methodological challenges of conducting robust quantitative research, particularly on vulnerable migrant populations, including the difficulty in accessing different groups of migrants, including undocumented migrants, or asylum seekers and refugees not based in camps or reception centres. With young migrants there is an additional complication of not being able to confirm ages in cases where identity documents are lacking, or in the case of very young migrants, not being able to get consent for conducting research in the absence of parents or guardians.

In terms of types of migrants, the focus of the included studies was primarily on asylum seekers and refugees, and particularly on unaccompanied migrant children, as well as irregular migrants. This means that our understanding of vulnerabilities to sexual violence of migrants not falling into any of these categories is largely absent. This is problematic as there is evidence that certain groups of young migrants may be even more vulnerable to sexual violence, such as LGBTQ+ migrants [3,16,19]. There is also some evidence that different groups of young migrants may be more vulnerable to certain forms of sexual violence; for example, young male migrants may be more vulnerable to sexual exploitation to meet basic needs while young female migrants may be more vulnerable to intimate partner violence [6]. There are also indications that sexual violence perpetrated against male migrants tends to be more physically violent than that against female migrants [3].

The lack of consensus on definitions and their operationalisation has been raised by Jud and colleagues [7] in relation to all kinds of violence against ‘children in migration’ and by Tan and Kuschminder [3] in relation to GBV and migration, and this was further supported by this review. Different types of violence were not always analysed separately, conflating sexual violence with other forms of GBV including emotional, physical and socioeconomic violence. Although there is some overlap, there are also large differences particularly in terms of potential perpetrators as well as in terms of health outcomes, particularly mental health outcomes. The behaviours that are included in the definition of sexual violence and how they are measured will also impact the rates that are found [7]. For example, the Longobardi et al. [20] study defined sexual touching as touching of genitals or breasts and any other touching would be excluded by this definition. These different definitions of sexual violence also hamper a comparison of results [7] as they measure slightly different phenomena.

The danger of this lack of evidence is highlighted by Digidiki & Bhabha [23] where they refer to ‘the institutional inability to acknowledge the phenomenon and the resulting false perception that a lack of evidence implies its nonexistence’. In other words, a ‘no evidence equals no problem’ approach, resulting in that victim identification, prevention and treatment of sexual violence are considered a low priority need, creating a vicious cycle of continued lack of attention to this issue [23]. This lack of attention leaves young migrants continually vulnerable to sexual violence while simultaneously not addressing the barriers to prevention and care services.

Despite the many research challenges, it is clear that young migrants, both male and female, are at risk of and do experience sexual violence. It is also increasingly evident that young migrants are vulnerable to sexual violence at all stages of migration [29,30] and that there are multiple sources of vulnerabilities at every level of the socioecological model. In line with previous research, this review points to the critical role of structural factors contributing to the vulnerabilities of young migrants to sexual violence [6,29]. These structural factors can facilitate access to safe and appropriate accommodations and mitigate the effects of economic hardship and enable access to trustworthy adults. On the other hand, these structural factors could also contribute to increased risk of sexual violence. For example, legislative mechanisms, such as closing of borders into Europe and within Europe, increasing migrants’ vulnerabilities to predatory persons and people smugglers. This also includes policy aspects, such migration policies, social welfare policies and healthcare policies, limiting young migrants’ access to legal and social rights in their host countries, including services for sexual violence [3,6,31].

Although it is challenging to draw definitive conclusions regarding the various forms of sexual violence to which young migrants are exposed, the existing research does lend support to the notion that such exposure tends to be ‘gender-balanced’ [3,26,30], both young male and female migrants are vulnerable to sexual violence. It could also reflect the larger number of young men as compared to young women who migrate as asylum seekers and refugees, two groups potentially more vulnerable to sexual violence than other types of migrants [3].

### Recommendations and ways forward

Regarding sexual violence that has taken place in the countries of origin and in transit, it is important that victims are identified and that they can access treatment to deal with the physical and mental health consequences of their experiences, regardless of their legal status. Regarding sexual violence that takes place in Europe, it is increasingly critical that host countries act decisively to prevent further experiences of sexual violence and that these actions target all levels of the socioecological model. At the individual and relationship levels this includes enabling young migrants’ participation in the decisions that affect them. It also involves enabling them to feel that they are working towards their own goals whether these be migration, education or employment goals. It also includes having access to safe and secure accommodation and trustworthy adults [6]. At the more structural level it includes enabling access to basic human rights and welfare services [3,6].

It is also critical to focus on the experience of young people in particular. Studies that focus only on children, especially those carried out in host countries, might miss the heightened vulnerabilities of young asylum seekers and undocumented migrants who have turned 18 and from one day to the next lose access to many rights and services they had as minors, including access to many healthcare and social services [6].

### Methodological considerations

The iterative nature of critical interpretive synthesis which facilitates the flexible nature of this review also means that some parts of the process may be more difficult to reproduce [17]. To mitigate this and to enhance internal validity we have been detailed regarding both the methods and considerations of the results.

A strength of this review is the inclusive nature of definitions of both sexual violence and migration allowing for a broader inclusion of studies in this review. However, studies focusing on human trafficking were excluded which means that some findings related to trafficking for the purpose of sexual exploitation were not included. This decision was done to avoid another term lacking consensus, which could introduce an additional layer of uncertainty to our results. Trafficking can encompass various forms of violence experienced by trafficked individuals, including sexual violence, and which may or may not occur across borders. However, trafficked individuals may be represented in the included studies. Although international students and expatriates were not included in the original search terms, these searches were carried out at a later date and no additional studies were identified for inclusion.

## Conclusion

There is a dearth of evidence on the vulnerabilities to and experiences of sexual violence among young migrants in Europe. This lack of evidence stems from a number of factors including a lack of consensus regarding critical terms employed in such research including sexual violence, migrants, and who classifies as a young person as well as methodological difficulties in conducting the research. However, it is critical to try to reach a consensus and overcome these challenges as what little evidence there is points to a serious and largely invisible problem that is prevalent among young migrants. Having appropriate evidence would enable appropriate prevention and response strategies to be put in place to protect and care for these young persons and facilitate their integration and participation in society.

## In addition

TAN was primarily responsible for the study design, conducted the literature search, undertook the title and abstract review, full-text review, conducted the data extraction and drafted the manuscript.

TH Conducted the full-text review

BOA Conducted the abstract review

## Appendix – Detailed Search Strategy

**PubMed**

#1

(”Sex Offenses”[Mesh]) OR (”sexual violence” OR ”sexual abuse” OR ”sex abuse” OR ”sexual trauma” OR ”sexual assault” OR ”sex crimes” OR ”sexual consent” OR rape)

n= 42,660

#2

((”Emigrants and Immigrants”[Mesh]) OR (”Transients and Migrants”[Mesh])) OR (emigrants OR immigrants OR migrants OR refugees OR ”asylum seekers”)

n= 97,648

#3

((”Adolescent”[Mesh]) OR ”Young Adult”[Mesh]) OR (youth OR teenager OR adolescent OR adolescents OR ”young person” OR girls OR boys OR child OR children)

n=5,822,400

#4

(((”Sex Offenses”[Mesh]) OR (”sexual violence” OR ”sexual abuse” OR ”sex abuse” OR ”sexual trauma” OR ”sexual assault” OR ”sex crimes” OR ”sexual consent” OR rape)) AND (((”Transients and Migrants”[Mesh]) OR ”Emigrants and Immigrants”[Mesh]) OR (emigrants OR immigrants OR migrants OR refugees OR ”asylum seekers”))) AND (((”Adolescent”[Mesh]) OR ”Young Adult”[Mesh]) OR (youth OR teenager OR adolescent OR adolescents OR ”young person” OR girls OR boys OR child OR children))

n=616

Filters:

- English language
- Published after 2002.01.01

n= 532

**Web of Science**

#1

(((((TS=(sex offences)) OR TS=(sexual violence)) OR TS=(sexual abuse)) OR TS=(sexual trauma)) OR TS=(sex crimes)) OR TS=(rape)

n=169,325

#2

((((TS=(migrant)) OR TS=(emigrant)) OR TS=(immigrant)) OR TS=(refugee)) OR TS=(asylum seeker)

n= 246,342

#3

(((((((TS=(adolescent)) OR TS=(young adult)) OR TS=(young person)) OR TS=(young people)) OR TS=(youth)) OR TS=(teenager)) OR TS=(girl)) OR TS=(boy)

n= 4,101954

#4

(((((TS=(sex offences)) OR TS=(sexual violence)) OR TS=(sexual abuse)) OR TS=(sexual trauma)) OR TS=(sex crimes)) OR TS=(rape) AND ((((TS=(migrant)) OR TS=(emigrant)) OR TS=(immigrant)) OR TS=(refugee)) OR TS=(asylum seeker) AND (((((((TS=(adolescent)) OR TS=(young adult)) OR TS=(young person)) OR TS=(young people)) OR TS=(youth)) OR TS=(teenager)) OR TS=(girl)) OR TS=(boy)

N=1015

Filters:

- Document type: articles & review articles
- English
- From 2002.01.01

N=855

**CINAHL**

#1

MH Rape OR MH Sexual abuse OR sex offences OR sexual violence OR sexual abuse OR sex abuse OR sexual trauma OR sexual assault OR sex crimes OR rape

n=43925

#2

MH (transients and migrants) OR MH immigrants OR MH refugee OR migrants OR emigrants OR immigrants OR asylum seekers

n=36,783

#3

MH adolescents OR MH young adults OR MH children OR youth OR teenager OR young person OR young people OR girls OR boys OR child OR adolescents OR adolescent

n=1,005,883

#4

MH Rape OR MH Sexual abuse OR sex offences OR sexual violence OR sexual abuse OR sex abuse OR sexual trauma OR sexual assault OR sex crimes OR rape AND MH (transients and migrants) OR MH immigrants OR MH refugee OR migrants OR emigrants OR immigrants OR asylum seekers AND MH adolescents OR MH young adults OR MH children OR youth OR teenager OR young person OR young people OR girls OR boys OR child OR adolescents OR adolescent

n=339

Filters:

- Academic journals
- English language
- From 2002

n= 309
